# Molecular Identification of a Novel Hantavirus in Malaysian Bronze Tube-Nosed Bats (*Murina aenea*)

**DOI:** 10.3390/v11100887

**Published:** 2019-09-21

**Authors:** Brigitta Zana, Gábor Kemenesi, Dóra Buzás, Gábor Csorba, Tamás Görföl, Faisal Ali Anwarali Khan, Nurul Farah Diyana Ahmad Tahir, Safia Zeghbib, Mónika Madai, Henrietta Papp, Fanni Földes, Péter Urbán, Róbert Herczeg, Gábor Endre Tóth, Ferenc Jakab

**Affiliations:** 1 Szentágothai Research Centre, Virological Research Group Pécs Hungary, University of Pécs, 7624 Pécs, Hungary; brigitta.zana@gmail.com (B.Z.); dora.buzas@gmail.com (D.B.); zeghbib.safia@gmail.com (S.Z.); mmoni84@gmail.com (M.M.); phencsi@gmail.com (H.P.); fanni4444@gmail.com (F.F.); toth.gabor.endre@gmail.com (G.E.T.); 2 Institute of Biology, Faculty of Sciences, University of Pécs, 7622 Pécs, Hungary; urpe.89@gmail.com; 3 Department of Zoology, Hungarian Natural History Museum, 1083 Budapest, Hungary; csorba.gabor@nhmus.hu (G.C.); gorfol.tamas@nhmus.hu (T.G.); 4 Faculty of Resource Science and Technology, UniversitiMalaysia Sarawak, Kota Samarahan 94300, Malaysia; fanwaral@gmail.com (F.A.A.K.); farahdiyanatahir@gmail.com (N.F.D.A.T.); 5 Microbial Biotechnology Research Group, Szentágothai Research Centre, University of Pécs, 7624 Pécs, Hungary; 6 Szentágothai Research Centre, Bioinformatics Core Facility, Bioinformatics Research Group, University of Pécs, 7624 Pécs, Hungary

**Keywords:** Sarawak mobatvirus, MinION, Tb1-Lu, *Mobatvirus*, one health concept

## Abstract

In the past ten years, several novel hantaviruses were discovered in shrews, moles, and bats, suggesting the dispersal of hantaviruses in many animal taxa other than rodents during their evolution. Interestingly, the coevolutionary analyses of most recent studies have raised the possibility that nonrodents may have served as the primordial mammalian host and harboured the ancestors of rodent-borne hantaviruses as well. The aim of our study was to investigate the presence of hantaviruses in bat lung tissue homogenates originally collected for taxonomic purposes in Malaysia in 2015. Hantavirus-specific nested RT-PCR screening of 116 samples targeting the L segment of the virus has revealed the positivity of two lung tissue homogenates originating from two individuals, a female and a male of the *Murina aenea* bat species collected at the same site and sampling occasion. Nanopore sequencing of hantavirus positive samples resulted in partial genomic data from S, M, and L genome segments. The obtained results indicate molecular evidence for hantaviruses in the *M. aenea* bat species. Sequence analysis of the PCR amplicon and partial genome segments suggests that the identified virus may represent a novel species in the *Mobatvirus* genus within the Hantaviridae family. Our results provide additional genomic data to help extend our knowledge about the evolution of these viruses.

## 1. Introduction

Hantaviruses (Hantaviridae) cause two types of life-threatening human diseases, haemorrhagic fever with renal syndrome (HFRS) in Eurasia and hantavirus cardiopulmonary syndrome (HCPS) in the Americas [1]. To date, as a consensus, wild rodents were believed to be the natural hosts of hantaviruses. However, recent studies described several novel hantaviruses in shrews, moles, and bats, suggesting the dispersal of hantaviruses in several animal taxa during their evolution [2]. Following the first detection of hantaviruses in the *Nycteris hispida* (Nycteridae) and *Neoromicia nanus* (Vespertilionidae) bat species [3,4], eight bat-borne hantaviruses were described in different bat species from the Hipposideridae, Rhinolophidae, Emballonuridae, and Vespertilionidae families, and only one from the flying fox species Geoffroy’s rousette (*Rousettus amplexicaudatus*, Family: Pteropodidae) [5,6,7].

Phylogenetic analyses of most recent studies have raised the possibility that bats or other animals (shrews and moles) of the Laurasiatheria superorder may have served as the primordial mammalian host, and harboured the ancestors of rodent-borne hantaviruses [5,7]. However, complex analyses for the genetic diversity and phylogeography of bat-associated hantaviruses are tentative, since complete genomic data is available only from *Brno virus* (BRNV), *Dakrong virus* (DKGV), *Láibīn virus* (LAIV), *Quezon virus* (QZNV), and *Xuân Sơn virus* (XSV). Unfortunately, in the case of other bat-associated hantaviruses just partial genomic fragments are available, mainly from the conservative L segment, hardening the implementation of evolutionary analyses [5,6,8].

Here, we present a presumably novel hantavirus tentatively named *Sarawak mobatvirus* (SARV) within the *Mobatvirus* genus detected in Bronze tube-nosed bats (*Murina aenea*, Vespertilionidae) from Malaysia. Our partial sequences from the S, M and L segments may contribute to getting closer to understanding the evolution of hantaviruses, and provide an important milestone of hantavirus research in Malaysia.

## 2. Materials and Methods

### 2.1. Sample Collection and Nucleic Acid Extraction

**Figure 1:**
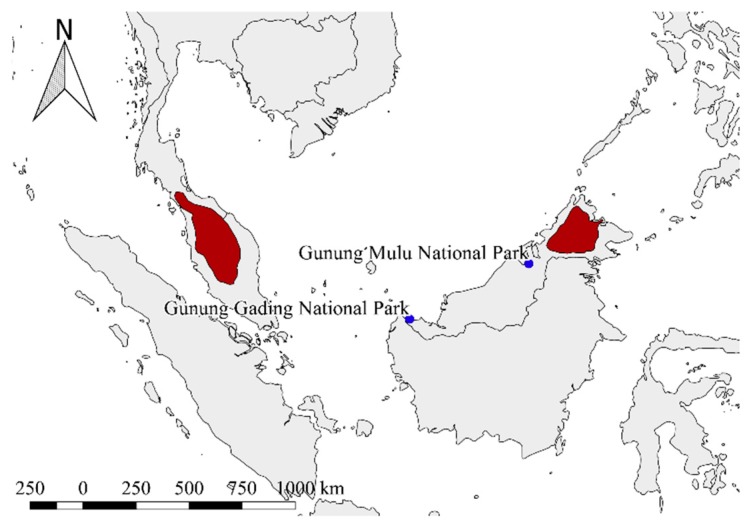
The distribution area of Bronze Tube-nosed Bat (*Murina aenea*) according to the IUCN (International Union for Conservation of Nature) Red List database is marked with red colour. Collection sites for the study are marked with blue dots.

Lung tissue samples were collected in Gunung Mulu National Park and Gunung Gading National Park, Malaysia in 2015 (Figure 1). Bats were captured by mist nets and harp traps in their foraging habitats. Species identification was performed with morphologic keys supported by the works of Francis (2008) and Hill (1964) [9,10], along with our own unpublished identification key, using the comparative materials housed in the Natural History Museum, London and the Hungarian Natural History Museum, Budapest. Depending on species identification and ethical consideration, selected individuals were subjected to museum-type collection and were dissected on site. Tissue samples were stored and transported in a Dry Shipper containing liquid nitrogen. Total RNA was extracted from lung tissues using the QIAamp MinElute Virus Spin Kit (Qiagen, Hilden, Germany). The field sampling was carried out according to widely approved ethical guidelines of handling mammalian species [11] with research permissions NCCD.907.4.4(JLD.12)-168 (Date of approval: 2015.10.29.), 422/2015 (Date of approval: 2015.10.29.) and DF.945.201(Jrd.5)-62 (Date of approval: 2016.01.07.), and export permit no. 16024 (Date of approval: 2016.02.19.), issued by the Controller of National Parks and Nature Reserves, Forest Department, Sarawak, Malaysia.

### 2.2. PCR Screening

All samples were subjected to a nested reverse transcription-PCR (RT-PCR) system using previously published, hantavirus-specific, universal degenerated nested primer sets [12]. First round PCR reactions were performed with the OneStep RT-PCR Kit (Qiagen) with the following cycling conditions: reverse transcription at 50 °C for 30 min and 95 °C for 15 min, then 40 cycles of denaturation at 94 °C for 1 min, annealing at 53 °C for 30 s, elongation at 72 °C for 1 min, and final elongation at 72 °C for 10 min. Second round PCR reactions were performed using the GoTaq G2 Flexi DNA Polymerase Kit (Promega, Madison, WI, USA) with an initial denaturation at 95 °C for 5 min, 40 cycles of denaturation at 94 °C for 1 min, annealing 52 °C for 45 s, elongation at 72 °C for 1 min, and final elongation at 72 °C for 10 min. PCR products were sequenced bidirectionally using the BigDye Terminator v1.1 Cycle Sequencing Kit (Applied Biosystems, Foster City, CA, USA) on the ABI Prism 310 genetic analyzer platform (Applied Biosystems).

### 2.3. Metagenomic cDNA Preparation and Nanopore Sequencing

Prior to Nanopore sequencing, lung tissue homogenates were exposed to enrichment protocol as detailed previously [13], followed by a ribodepletation procedure using The RiboMinus™ Eukaryote System v2 kit (Life Technologies. Complementary DNA (cDNA)). Samples were then subjected to the Sequence Independent Single Primer Amplification (SISPA) approach [14]. cDNA amplification was performed by an AMV Reverse Transcriptase (Promega) according to the provided manual by the manufacturers using the FR26RV-N (5′-GCCGGAGCTCTGCAGATATCNNNNNN-3′) primer. Thereafter, ds cDNA was amplified by a DreamTaq DNA Polymerase (Thermo Fisher Scientific, Waltham, MA, USA) according to the supplied protocol using the FR20RV (5′-GCCGGAGCTCTGCAGATATC-3′) primer. Amplified cDNA was purified by NucleoSpin^®^ Gel and a PCR Clean-up kit (Macherey-Nagel, Düren, Germany), and quantified using a Qubit dsDNA BR Assay kit (Thermo Fisher Scientific). Libraries were prepared using the PCR Barcoding Kit (SQK-PBK004) and protocol by Oxford Nanopore Technologies. R9.4.1 flow cell and MinKNOW v2.0 software were used for sequencing. As the first step of sequence data analysis, adapters were trimmed using Porechop v0.2.4 [15], with default settings. Reads with internal adapters, which were indicating chimaera reads, were also split (--midle_treshold) with Porechop. For long read alignment, DIAMOND was used against the full NCBI NR (National Center for Biotechnology Information non-redundant protein) database with the following options turned on: -F 15 --range-culling [16]. *Mobatvirus*-related sequences were extracted from the DIAMOND results, and we used these sequences for further analysis.

### 2.4. In vitro Virus Propagation

*Tadarida brasiliensis* lung tissue cells (Tb1-Lu, ATCC^®^ CCL-88™) (Lonza, Basel, Switzerland) and Vero E6 kidney cells (ATCC^®^ CRL-1586™) were maintained in EMEM and DMEM (Lonza, Switzerland) respectively, supplemented with 10% Fetal Bovine Serum (Biosera, Nuaillé, France) and 1% Penicillin-Streptomycin (Lonza, Switzerland) at 37 °C with 5% CO_2_ until at 70% confluency in a 24-well plate. An amount of 200 µL of supernatant from each hantavirus PCR positive lung tissue homogenate was placed on the cell monolayer and incubated for 1 h at 37 °C. Thereafter, cells were supplemented with 1 mL of extra fresh medium and were monitored for cytopathogenic effect for 7 days postinfection. After 7 days, cells were frozen at −80 °C and thawed in order to lyse the cells, and 200 µL of the inoculums was used for every 7 additional passages from the previous plates.

### 2.5. Phylogenetic Analyses

Phylogenetic tree reconstruction was implemented using the MrBayes v3.2.4 software, with the GTR+G+I substitution model. Analysis settings were as follows: 10 million generations (25% discarded as burn-in), sampled every 1000 generations. The run was stopped after three and a half million generations when the standard deviation of split frequencies was 0.003. Trees were edited using the iTol online tool [17].

## 3. Results and Discussion

### 3.1. Virus Detection

Hantavirus-specific nested RT-PCR screening was carried out on 116 bat lung tissue samples representing 9 bat families, 15 genera, and 33 species (Table S1). Hantavirus RNA was detected in two Bronze Tube-nosed Bat (*M. aenea*) lung tissue samples from Gunung Mulu National Park, Malaysia (Figure 1). These two samples were collected from one male and one female *M. aenea* bat at the same sampling occasion. Unfortunately, all attempts to amplify the complete genome of this hantavirus failed, despite the fact we applied a combination of multiple primers (Table 1) as was previously described [18,19]. The attempts to isolate the SARV on the Tb1-Lu and VeroE6 cell lines failed as well. After the 7th blind passage, we could not detect hantavirus RNA in the supernatants of lysed cells by the previously mentioned nested RT-PCR system. The failure of complete genome amplification and isolation of the virus may be due to many factors, such as the high-level diversity of hantavirus sequences carried by bats, low virus titer in the examined organ, or virus RNA degradation as a consequence of improper tissue preservation as is suggested by previous studies [4,5]. Despite this, in vitro isolation of rodent-borne hantaviruses is well established on Vero E6 cells, but newly identified hantaviruses carried by bats, insectivores, and rodents may remain uncultured, probably due to the lack of host-derived cell lines [20]. Nanopore sequencing of SARV positive samples has revealed partial genome sequences of 671, 1326, and 677 nucleotides of the S, M, and L segments, respectively. To date, our results give the first molecular evidence of the presence of bat-related hantaviruses in the country, although mostly serological data are available, suggesting the presence of hantaviruses other than *Mobatviruses* in Malaysia [21,22,23].

### 3.2. Sequence and Phylogenetic Analysis

Pairwise alignment of partial S, M, and L sequences of SARV (GenBank: MN337866-70) were compared to strains from *Orthohantavirus*, *Mobatvirus*, *Loanvirus,* and *Thottimivirus* genera available in GenBank (Figure S1; Table S2). The partial S (671 nucleotides) and partial L (383 nucleotides) sequences of SARV displayed 85.7% and 92.1% amino acid sequence similarity respectively against a *Laibin virus* strain (GenBank: MK393932) from Myanmar. The partial M (1326 nucleotides) sequence of SARV displayed 84.1% amino acid sequence similarity to another *Laibin virus* strain (GenBank: MK064115) from Myanmar. According to the currently recognized demarcation criterion of International Committee on Taxonomy of Viruses (ICTV), a 7% difference in the amino acid levels of the nucleocapsid protein (NP) and glycoprotein precursor (GPC) is the limit to assign a hantavirus as a tentatively novel species. Although the complete genome could not be amplified, based on the sequence homology data we assume that SARV may represent a new hantavirus species within the *Mobatvirus* genera.

**Figure 2:**
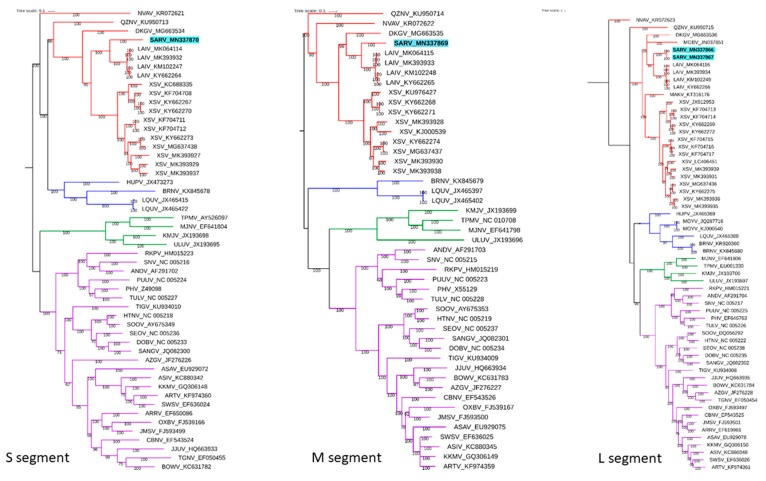
Maximum likelihood analysis of hantavirus S (671 nucleotides), M (1326 nucleotides), and L (383 nucleotides) segments respectively, visualized by the iTol online server. *Sarawak mobatvirus* reported in this study is highlighted with a blue background and deposited in GenBank under the Accession numbers of MN337866-70. Branch colours for *Mobatviruses* are highlighted with red, *Loanviruses* are highlighted with blue, *Thottimiviruses* are highlighted with green, and *Orthohantaviruses* are highlighted with purple.

Maximum likelihood phylogenetic analysis of S, M, and L segments of members of *Orthohantavirus*, *Mobatvirus*, *Loanvirus,* and *Thottimivirus* (Table S2) genera has revealed SARV segregated into the clade of *Mobatviruses* where SARV is displaying a monophyletic group with *Laibin virus*. Analyses of all segments suggest that SARV shared a common ancestor with *Laibin virus* which is its currently known closest relative (Figure 2). To date, several hantaviruses belonging to the *Loanvirus* genus have been described inVespertilionid bats (*Brno virus, Mouyassué virus*, and *Huangpi virus*) [5], but *Sarawak mobatvirus* represents the first member of the *Mobatvirus* genus described in the Vespertilionid bat *M. aenea*.

## 4. Conclusions

Among mammals, bats (Chiroptera) represent the second most diverse order with approximately 1400 species distributed throughout the world except the arctic areas [24]. To date, several evolutionary studies have confirmed that bats represent the most ancestral hosts of many viruses and they are able to asymptomatically host a range of viruses including highly pathogenic zoonotic agents for humans and animals [25,26]. In past decades, the increasing discovery of hantaviruses in bats has raised the possibility that bats may be the primordial host of ancient hantaviruses [5].

The tentatively novel member of the *Mobatvirus* genus, SARV, was identified from a rare bat species, *M. aenea* (Vespertilionidae). This species has a limited distribution restricted to the Malayan Peninsula and Borneo [27]. The bats caught in the Gunung Mulu National Park represent new, unpublished records of the species, and highlight the importance of studies on the bats of lowland primary forests following the concept of OneHealth studies. Although no direct observation about the roosting behaviour of *M. aenea* bat species has been published, known capture sites (being exclusively in forests and never in caves) [9,10,28] and the distinctive colouration typical for bats hanging free among leaves strongly indicate the foliage-dweller roost type. This roosting means a highly reduced chance of physical contact among different species as compared with cave-dweller bats, where mixed-species colonies are frequent [29,30]. This latter information strengthens the hypothesis that bats served as primordial hosts of ancient hantaviruses, since the chance for spillover events connected to this species in the past is very low.

## Figures and Tables

**Table 1:** Oligonucleotide primers used to amplify the complete S, M, and L segments of *Sarawak mobatvirus*.

Genomic Segment	Primer Name	Primer Sequence (5′–3′)	Reference
S, M, L	OSM55F	TAGTAGTAGACTCC	[18]
S	HVSF1	TAGTAGTAGACTCCTTRAARAGC	[19]
	HVSR1906	TAGTAGTAKRCWCCYTRARAAG	
M	HVMF1	TAGTAGTAGACWCCGCAAAAG	
	HVMR3684	TAGTAGTATRCTCCGCARG	
L	HVLF1	TAGTAGTAGACTCCRGA	
	HVLR6561	TAGTAGTAGTAKRCTCCGRGA	

## Supplementary Materials

The following are available online at https://www.mdpi.com/1999-4915/11/10/887/s1, Figure S1: Pairwise alignment of S, M and L segments of viruses in Hantaviridae family compared to Sarawak mobatvirus. Our sequence is indicated with S_segment, M_segment and L_segment titles, Table S1: List of bat species examined in this study, Table S2: Hantavirus accession number list used for pair-wise alignment and phylogenetic analysis.
