# Supplementary material for: Molecular Identification of a Novel Hantavirus in Malaysian Bronze Tube-Nosed Bats (Murina aenea)
Source: Viruses. 2019 Sep 21;11(10):887. doi: 10.3390/v11100887 (PMC6832519; doi:10.3390/v11100887)
Supplement: Supplementary file 1 [file viruses-11-00887-s001.pdf]

viruses

MDPI

Supplemetary Materials

Table S1: List of bat species examined in this study

Family	Genus	Species	Common name
Emballonuridae	*Saccolaimus*	*saccolaimus*	Bare-rumped Sheathtail-bat
Hipposideridae	*Hipposideros*	*ater*	Dusky Leaf-nosed Bat
Hipposideridae	*Hipposideros*	*cervinus*	Fawn-colored Leaf-nosed Bat
Hipposideridae	*Hipposideros*	*diadema*	Diadem Leaf-nosed Bat
Hipposideridae	*Hipposideros*	*dyacorum*	Dayak Leaf-nosed Bat
Hipposideridae	*Hipposideros*	*galeritus*	Cantor's Leaf-nosed Bat
Hipposideridae	*Hipposideros*	*larvatus*	Horsfield's Leaf-nosed Bat
Megadermatidae	*Megaderma*	*spasma*	Lesser False Vampire
Miniopteridae	*Miniopterus*	*australis*	Little Long-fingered Bat
Molossidae	*Chaerephon*	*plicatus*	Wrinkle-lipped Free-tailed Bat
Nycteridae	*Nycteris*	*tragata*	Malayan Slit-faced Bat
Pteropodidae	*Balionycteris*	*maculata*	Spotted-winged Fruit Bat
Pteropodidae	*Cynopterus*	*brachyotis*	Lesser Dog-faced Fruit Bat
Rhinolophidae	*Rhinolophus*	*affinis*	Intermediate Horseshoe Bat
Rhinolophidae	*Rhinolophus*	*borneensis*	Bornean Horseshoe Bat
Rhinolophidae	*Rhinolophus*	*creaghi*	Creagh's Horseshoe Bat
Rhinolophidae	*Rhinolophus*	*philippinensis*	Large-eared Horseshoe Bat
Rhinolophidae	*Rhinolophus*	*trifoliatus*	Trefoil Horseshoe Bat
Vespertilionidae	*Glischropus*	*tylopus*	Common Thick-thumbed Bat
Vespertilionidae	*Kerivoula*	*hardwickii*	
Vespertilionidae	*Kerivoula*	*minuta*	Least Woolly Bat
Vespertilionidae	*Kerivoula*	*papillosa*	Papillose Woolly Bat
Vespertilionidae	*Kerivoula*	*pellucida*	Clear-winged Woolly Bat
Vespertilionidae	*Murina*	*rozendaali*	Gilded Tube-nosed Bat
Vespertilionidae	*Murina*	*aenea*	Bronze Tube-nosed Bat
Vespertilionidae	*Myotis*	*horsfieldi*	Horsfield's Myotis
Vespertilionidae	*Myotis*	*muricola*	
Vespertilionidae	*Myotis*	*ridleyi*	Ridley's Myotis
Vespertilionidae	*Phoniscus*	*atrox*	Groove-toothed Trumpet-eared Bat
Vespertilionidae	*Tylonycteris*	*pachypus*	Lesser Bamboo Bat

Figure S1: Pairwise alignment of S, M and L segments of viruses in *Hantaviridae* family compared to Sarawak mobatvirus. Our sequence is indicated with S_segment, M_segment and L_segment titles.

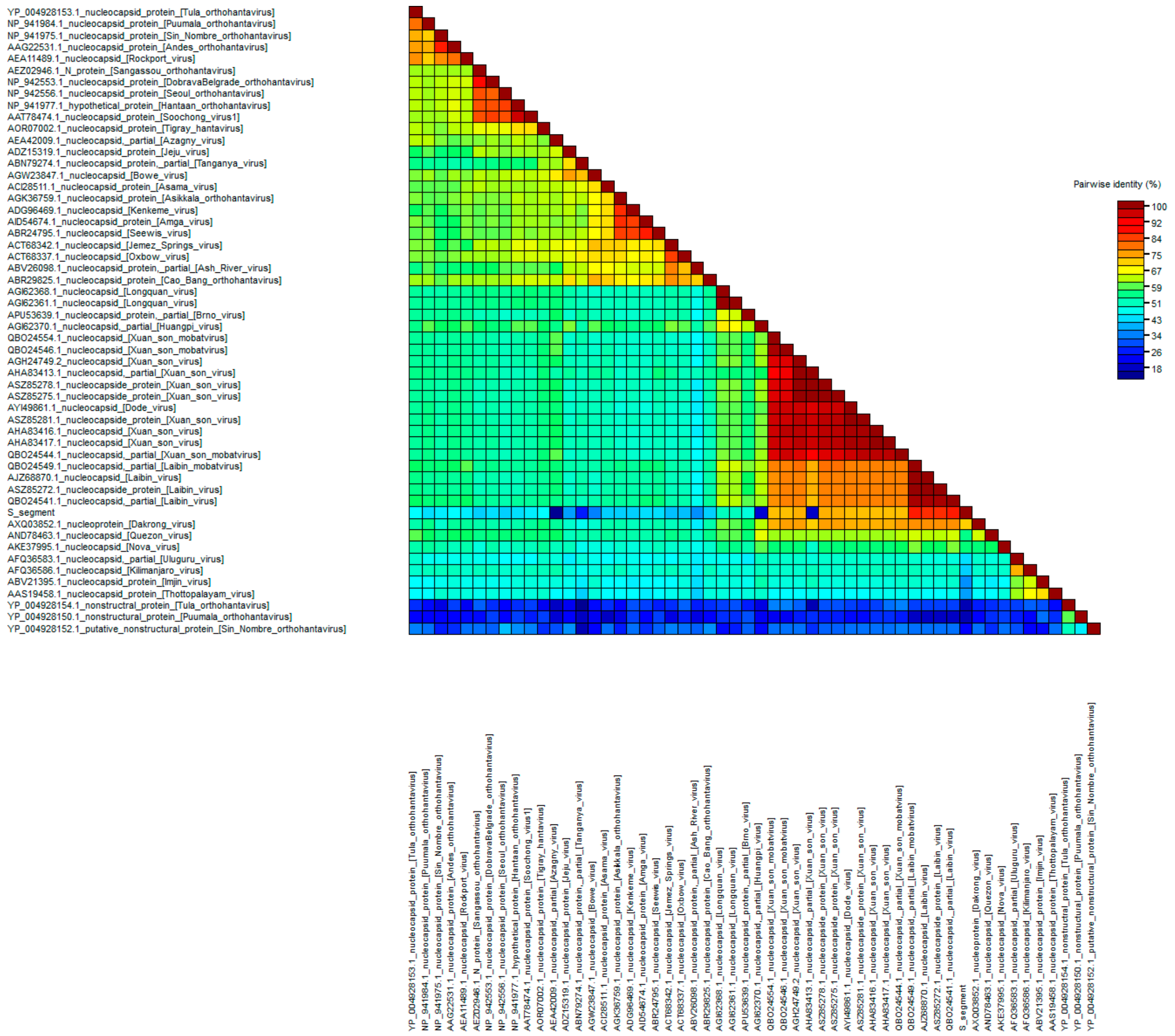

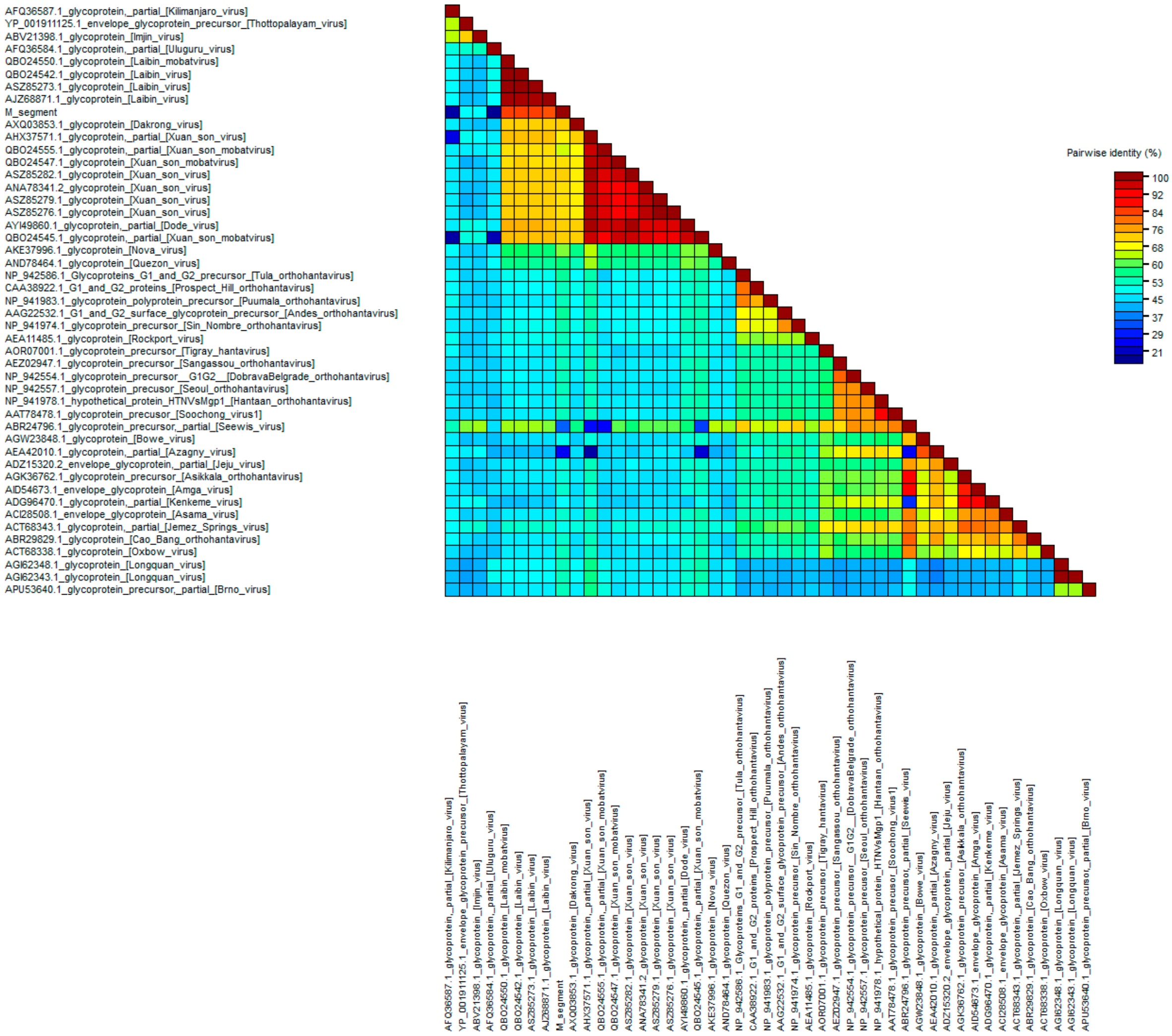
AFQ36587.1_glycoprotein,_partial_[Kilimanjaro_virus]
YP_001911125.1_envelope_glycoprotein_precursor_[Thottopalayam_virus]
ABV21398.1_glycoprotein_[Imjin_virus]
AFQ36584.1_glycoprotein,_partial_[Uluguru_virus]
QBO24550.1_glycoprotein_[Laibin_mobatvirus]
QBO24542.1_glycoprotein_[Laibin_virus]
ASZ85273.1_glycoprotein_[Laibin_virus]
AJZ68871.1_glycoprotein_[Laibin_virus]
M_segment
AXQ03853.1_glycoprotein_[Dakrong_virus]
AHX37571.1_glycoprotein,_partial_[Xuan_son_virus]
QBO24555.1_glycoprotein,_partial_[Xuan_son_mobatvirus]
QBO24547.1_glycoprotein_[Xuan_son_mobatvirus]
ASZ85282.1_glycoprotein_[Xuan_son_virus]
ANA78341.2_glycoprotein_[Xuan_son_virus]
ASZ85279.1_glycoprotein_[Xuan_son_virus]
ASZ85276.1_glycoprotein_[Xuan_son_virus]
AYI49860.1_glycoprotein,_partial_[Dode_virus]
QBO24545.1_glycoprotein,_partial_[Xuan_son_mobatvirus]
AKE37996.1_glycoprotein_[Nova_virus]
AND78464.1_glycoprotein_[Quezon_virus]
NP_942586.1_Glycoproteins_G1_and_G2_precursor_[Tula_orthohantavirus]
CAA38922.1_G1_and_G2_proteins_[Prospect_Hill_orthohantavirus]
NP_941983.1_glycoprotein_polyprotein_precursor_[Puumala_orthohantavirus]
AAG22532.1_G1_and_G2_surface_glycoprotein_precursor_[Andes_orthohantavirus]
NP_941974.1_glycoprotein_precursor_[Sin_Nombre_orthohantavirus]
AEA11485.1_glycoprotein_[Rockport_virus]
AOR07001.1_glycoprotein_precursor_[Tigray_hantavirus]
AEZ02947.1_glycoprotein_precursor_[Sangassou_orthohantavirus]
NP_942554.1_glycoprotein_precursor__G1G2__[DobravaBelgrade_orthohantavirus]
NP_942557.1_glycoprotein_precusor_[Seoul_orthohantavirus]
NP_941978.1_hypothetical_protein_HTNVsMgp1_[Hantaan_orthohantavirus]
AAT78478.1_glycoprotein_precusor_[Soochong_virus1]
ABR24796.1_glycoprotein_precursor,_partial_[Seewis_virus]
AGW23848.1_glycoprotein_[Bowe_virus]
AEA42010.1_glycoprotein,_partial_[Azagny_virus]
ADZ15320.2_envelope_glycoprotein,_partial_[Jeju_virus]
AGK36762.1_glycoprotein_precursor_[Asikkala_orthohantavirus]
AID54673.1_envelope_glycoprotein_[Amga_virus]
ADG96470.1_glycoprotein,_partial_[Kenkeme_virus]
ACI28508.1_envelope_glycoprotein_[Asama_virus]
ACT68343.1_glycoprotein,_partial_[Jemez_Springs_virus]
ABR29829.1_glycoprotein_[Cao_Bang_orthohantavirus]
ACT68338.1_glycoprotein_[Oxbow_virus]
AGI62348.1_glycoprotein_[Longquan_virus]
AGI62343.1_glycoprotein_[Longquan_virus]
APU53640.1_glycoprotein_precursor,_partial_[Brno_virus]
Pairwise identity (%)
100
92
84
76
68
60
53
45
37
29
21

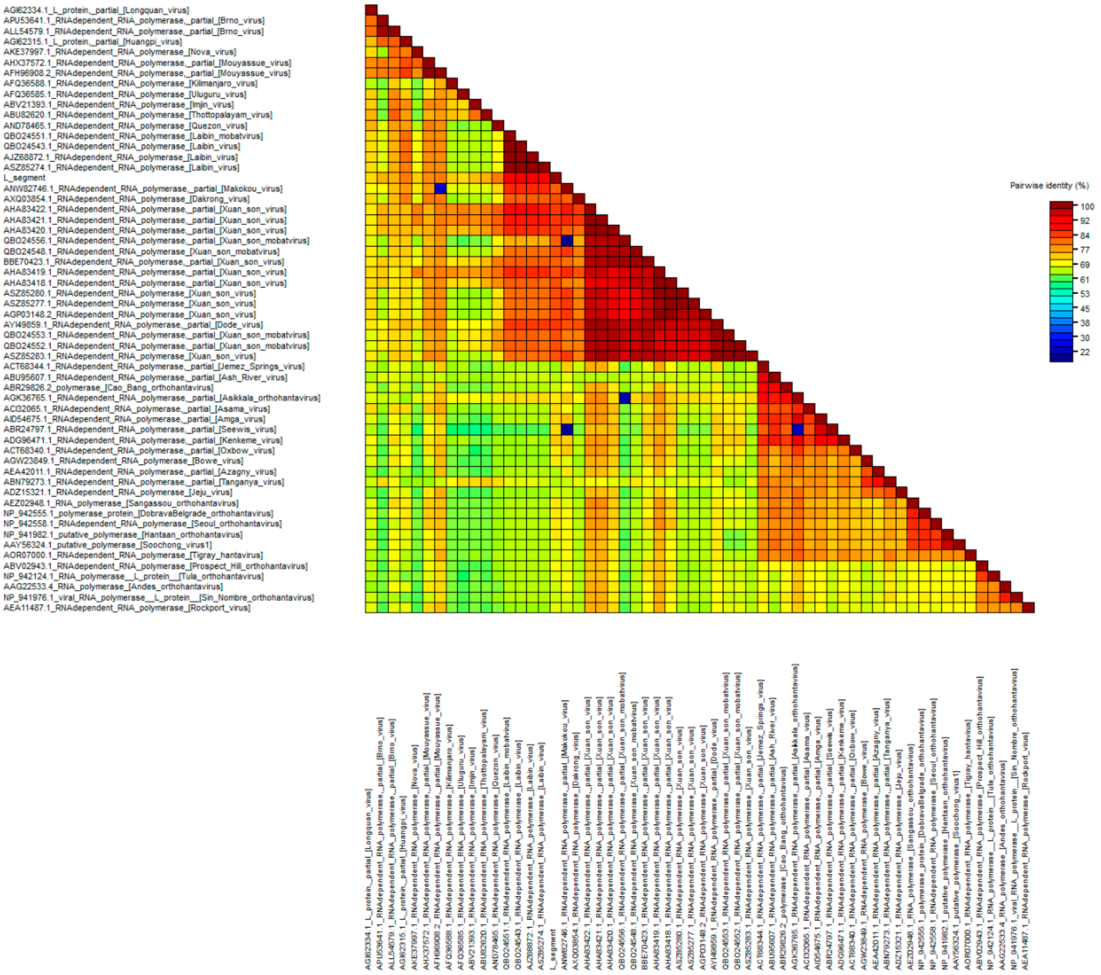

Table S2: Hantavirus accession number list used for pair-wise alignment and phylogenetic analysis

S segment:

KM102247; KY662264; MK064114; MK393932; KC688335; KF704708; KF704711; KF704712; KY662273; MG637438; MK393927; MK393929; MK393937; KY662267; KY662270; MG663534; JX473273; KX845678; JX465415; JX465422; KU950713; EF543524; EF650086; FJ593499; EF636024; KF974360; GQ306148; KC880342; AY526097; EF641804; JF276226; EF050455; KC631782; HQ663933; JX193695; JX193698; EU929072; FJ539166; HM015223; KR072621; NC_005216; AF291702; Z49098; NC_005227; NC_005224; NC_005233; NC_005218; AY675349; JQ082300; KU934010; NC_005236

M segment:

KM102248; KY662265; MK064115; MK393933; KU976427; KJ000539; KY662274; MG637437; MK393928; MK393930; MK393938; KY662268; KY662271; MG663535; KX845679; JX465397; JX465402; KU950714; EF543526; FJ593500; EF636025; KF974359; GQ306149; KC880345; NC_010708; EF641798; JF276227; KC631783; HQ663934; JX193696; JX193699; EU929075; FJ539167; KR072622; NC_005215; AF291703; X55129; NC_005228; NC_005223; NC_005234; NC_005219; AY675353; JQ082301; KU934009; NC_005237; HM015219

L segment

KM102249; KY662266; MK064116; MK393934; JX912953; KF704713; KF704714; KF704715; KF704716; KF704717; KY662275; MG637436; MK393935; MK393936; LC406451; MK393931; MK393939; KY662269; KY662272; MG663536; JN037851; JQ287716; KJ000540; JX465369; KX845680; KR920360; JX465388; KT316176; KU950715; EF543525; EF619961; FJ593501; EF636026; KF974361; GQ306150; KC880348; EU001330; EF641806; JF276228; EF050454; KC631784; HQ663935; JX193697; JX193700; EU929078; FJ593497; HM015221; KR072623; NC_005217; AF291704; EF646763; NC_005226; NC_005225; NC_005235; NC_005222; DQ056292; JQ082302; KU934008; NC_005238

Supplemetary
